# Decision trees to evaluate the risk of developing multiple sclerosis

**DOI:** 10.3389/fninf.2023.1248632

**Published:** 2023-08-15

**Authors:** Manuela Pasella, Fabio Pisano, Barbara Cannas, Alessandra Fanni, Eleonora Cocco, Jessica Frau, Francesco Lai, Stefano Mocci, Roberto Littera, Sabrina Rita Giglio

**Affiliations:** ^1^Department of Electrical and Electronic Engineering, University of Cagliari, Cagliari, Italy; ^2^Department of Medical Science and Public Health, Centro Sclerosi Multipla, University of Cagliari, Cagliari, Italy; ^3^Unit of Oncology and Molecular Pathology, Department of Biomedical Sciences, University of Cagliari, Cagliari, Italy; ^4^Medical Genetics, Department of Medical Sciences and Public Health, University of Cagliari, Cagliari, Italy; ^5^Centre for Research University Services, University of Cagliari, Monserrato, Italy; ^6^AART-ODV (Association for the Advancement of Research on Transplantation), Cagliari, Italy; ^7^Medical Genetics, R. Binaghi Hospital, ASSL Cagliari, ATS Sardegna, Cagliari, Italy

**Keywords:** decision trees, human leukocyte antigen, immunogenetic risk markers, likelihood of multiple sclerosis development, machine learning, multiple classifier, multiple sclerosis

## Abstract

**Introduction:**

Multiple sclerosis (MS) is a persistent neurological condition impacting the central nervous system (CNS). The precise cause of multiple sclerosis is still uncertain; however, it is thought to arise from a blend of genetic and environmental factors. MS diagnosis includes assessing medical history, conducting neurological exams, performing magnetic resonance imaging (MRI) scans, and analyzing cerebrospinal fluid. While there is currently no cure for MS, numerous treatments exist to address symptoms, decelerate disease progression, and enhance the quality of life for individuals with MS.

**Methods:**

This paper introduces a novel machine learning (ML) algorithm utilizing decision trees to address a key objective: creating a predictive tool for assessing the likelihood of MS development. It achieves this by combining prevalent demographic risk factors, specifically gender, with crucial immunogenetic risk markers, such as the alleles responsible for human leukocyte antigen (HLA) class I molecules and the killer immunoglobulin-like receptors (KIR) genes responsible for natural killer lymphocyte receptors.

**Results:**

The study included 619 healthy controls and 299 patients affected by MS, all of whom originated from Sardinia. The gender feature has been disregarded due to its substantial bias in influencing the classification outcomes. By solely considering immunogenetic risk markers, the algorithm demonstrates an ability to accurately identify 73.24% of MS patients and 66.07% of individuals without the disease.

**Discussion:**

Given its notable performance, this system has the potential to support clinicians in monitoring the relatives of MS patients and identifying individuals who are at an increased risk of developing the disease.

## 1. Introduction

Multiple sclerosis (MS) is an autoimmune-mediated disorder that affects the central nervous system (CNS) and is the most common cause of neurological disability in young adults (Compston and Coles, 2008; Ghasemi et al., 2017). The main feature of this condition is the appearance of areas of demyelination in the cerebral white and gray matter. In addition, a series of other processes such as infiltration of inflammatory cells in the parenchyma, oligodendrocytes damage and axonal loss have been reported (Lucchinetti et al., 2011; Dendrou et al., 2015; Reich et al., 2018). This can result in autonomic and sensorimotor defects, visual disturbances, ataxia, fatigue, cognitive disorders, and emotional problems (Hafler, 2004; Compston and Coles, 2008). Although the disease often has no symptoms in its early stages, it inevitably leads to disability if left untreated (Rolak, 2003; Brownlee et al., 2017). Depending on the site of the neurologic lesions, clinical symptoms can vary greatly (Nylander and Hafler, 2012). Two main forms of MS have been distinguished based on clinical characteristics, disease course and etiology: primary progressive multiple sclerosis (PPMS) and relapsing-remitting multiple sclerosis (RRMS) (Klineova and Lublin, 2018).

According to the World Health Organization (WHO), over 2.8 million people worldwide were diagnosed with MS (World Health Organization [WHO], 2023) and the prevalence is expected to increase over time (Browne et al., 2014; Dobson and Giovannoni, 2019). Interestingly, MS prevalence rates vary by latitudinal gradient around the world, increasing up to 10-fold between the equator and 60° north and south parallel (Simpson et al., 2019; Sabel et al., 2021). In detail, North Europe and North America have a high prevalence (> 30/100,000), southern parts of the United States and Central America have a medium prevalence (5–30/100,000), and Asia and South America have a low prevalence (5/100,000) (Koch-Henriksen and Sørensen, 2010; Tullman, 2013). Nevertheless, Sardinia, the second largest island of Mediterranean Sea, stands out as an exception to this gradient, with one of the highest MS rates in the world (361/100,000 inhabitants) (Frau et al., 2021).

Given its relevant prevalence, MS represents a significant health and socioeconomic burden on society at both the individual and national levels (Battaglia et al., 2022). Due to its wide range of manifestations, debilitating nature and onset during the most productive and active years of patients’ lives, MS has profound effects on their physical, psychological, social and economic wellbeing (Amato et al., 2002; Ghasemi et al., 2017).

Unfortunately, no effective cure for the disease has been discovered to date, due to an incomplete understanding of the pathogenic mechanisms and underlying causes (Auletta et al., 2012; Reich et al., 2018). The likelihood for an individual to develop MS is strongly influenced by her or his ethnic background and family history of disease, suggesting that genetic susceptibility is a key determinant of risk. Over the last decade, genome-wide association studies (GWAs) have identified more than 230 loci associated with MS susceptibility (The International Multiple Sclerosis Genetics Consortium, and The Wellcome Trust Case Control Consortium 2, 2011; Mitrovič et al., 2018; International Multiple Sclerosis Genetics Consortium, 2019a,b). However, only a few of these loci, such as *IL2RA* (*rs2182410*), *CD58* (*rs2300747*), *TNFRSF1A* (*rs1800693*), *EVI5* (*rs6689470*), *RGS1* (*rs7535818*), have consistently shown associations in all GWAS studies (Andlauer et al., 2016). These single nucleotide polymorphisms (SNPs) often impact regulatory regions or key susceptibility genes involved in the innate immune response, inflammation, cell death regulation, or synaptic function. Indeed, the main signal genome-wide maps on the major histocompatibility complex (MHC) region with the strongest association with the class II region of the human leukocyte antigen (HLA) gene cluster and explains up to 10.5% of the genetic variance underlying risk (Gourraud et al., 2012; Miljković and Spasojević, 2013; Veroni and Aloisi, 2021).

It is well established that the polymorphic alleles of the HLA complex have a significant effect on the risk of developing MS. For example, HLA-DRB1*15 alleles, especially HLA-DRB1*15:01, are highly associated with MS. On the plus side, HLA-DRB1*03 and HLA-DRB1*04 are also highly associated with MS (Brynedal et al., 2007; Hollenbach and Oksenberg, 2015). These genes encode proteins that bind and present antigenic peptides, which are displayed on the surface of cells for recognition by T cells (Huppa and Davis, 2003; van den Elsen, 2011; Kelly and Trowsdale, 2019). Consequently, these molecules play an important role in both defense against pathogens and autoimmunity (Todd et al., 1988; Nepom and Erlich, 1991; Prentice et al., 2015; Seliger et al., 2017).

Besides their role in antigen presentation, Class I HLA ligands are also recognized by killer immunoglobulin-like receptors (KIRs) expressed on natural killer (NK) and CD4+ and CD8+ T cells (Pende et al., 2019; Duygu et al., 2021). The main function of these receptors is to reduce the lymphocyte activation and attenuate the innate killing capacity of NK cells (Hanson et al., 2022). NK cells express 15 unique KIR receptors that have different interactions with specific subtypes of HLA Class I molecules (Dubreuil et al., 2021). The interaction KIR/HLA results in either activation or inhibitory signals (Biassoni and Malnati, 2018). Given that *KIR* may also be expressed by CD4 T-cells, it is conceivable that KIR diversity can influence specific antibody production and thus also explain some HLA class II associations in MS.

Several studies have demonstrated that various *KIR* haplotypes may be protective against MS. In fact, different KIR profiles, are involved both immunoregulatory dysfunction and inflammatory processes underlying MS pathogenic mechanisms (Lorentzen et al., 2009; Fusco et al., 2010; García-León et al., 2011).

In a previous work, some of the authors of this paper demonstrated that the analysis based on the entropy of the HLA and KIR immunogenetic systems could be used to determine an individual’s risk to develop MS (Melis et al., 2019). The entropy-based risk test combines standard statistical methods to evaluate immunogenetic parameters, associated with MS, with the analysis of the entropy for measuring the global disorder status. The definition of entropy introduced by Shannon was used to study the complex genetic systems, such as HLA and KIR and their impact on immune response mechanisms; the formula to compute the entropy involves probabilities and, since there is no theoretical model to derive those values, the frequencies parameters appearing were obtained experimentally from the control group. First, the aforementioned method implies the calculation of two entropies for each person, one for the HLA haplotypes, that is calculated for each of the 16 possible HLA haplotypes and the other for the KIR genes, that is calculated only for 6 couples of inhibitory KIR genes. Then, to allow the comparison between controls and patients, two ratios *R*_*HLA*_ and *R*_*KIR*_ are calculated between the entropy of patients and the mean entropy of controls, for HLA haplotypes and KIR genes, respectively. Finally, the index used to assess the risk of each subject in the study is the total entropy ratio *R*_*tot*_, that is calculated as the mean between HLA’s entropy ratio (*R*_*HLA*_) and KIR’s entropy ratio (R_*KIR*_). Based on the total entropy ratio *R*_*tot*_ of patients and controls, it was possible to distinguish three entropy intervals corresponding to three degrees of risk *R* of developing MS (low, medium, high). When analyzing the HLA and KIR systems combined, the total entropy ratio was significantly higher, with a *p*-value of 0.002, in patients affected by RRMS compared to controls.

Taking into consideration the complexity of MS, the present study sought to devise a novel algorithm employing machine learning (ML) techniques based on individual HLA and KIR profiles of MS patients and healthy subjects. The primary goal of this new algorithm was to establish a predictive tool capable of discerning the likelihood of developing the disease, while also predicting the onset of the two clinical forms, RRMS or PPMS. The method combines the most common demographic risk factors, i.e., the gender, with the most important immunogenetic risk markers previously established (Melis et al., 2019). In particular, the alleles coding for the HLA class I and II molecules (HLA-A, -B, -C, -DRB1) and the KIR genes encoding the receptors of NK lymphocytes have been included in the input features of the ML model. The rationale behind limiting the analysis to only the *HLA* and *KIR* loci is twofold. Firstly, this approach allows us to directly compare the efficacy of the new ML predictive model with the entropy analysis employed in a previous study (Melis et al., 2019). Secondly, the decision to include the KIR genes in the analysis is supported by recent publications suggesting that NK cell-mediated immunity may actively regulate MS evolution through different combinations of KIR/HLA haplotypes (Kaur et al., 2013; Bettencourt et al., 2014; Shahsavar et al., 2016; Karimizadeh et al., 2020).

The proposed predictive model makes use of decision trees (DTs) (Mitchell, 1997; Breiman, 1998) which are a simple supervised ML classification method. DTs offer a simple and intuitive way to represent and interpret gathered data. The unique feature of their decision-making process has made them quite popular in a wide range of medical fields (Lyell et al., 2021). Moreover, differently from many ML techniques based on black box models, difficult to be interpreted, DTs are able to provide, as a result of the classification, comprehensible rules behind the decisions taken, usable by medical staff for prognosis purposes. For the sake of comparison, a classical Naïve Bayes (NB) model (Mitchell, 1997) has been implemented, as it is a common benchmark in classification problems.

## 2. Materials and methods

### 2.1. Patients and control recruitment

A cohort of 299 MS patients who were referred to the Sardinian Regional Government Centre for Diagnosis and Treatment of MS was enrolled. The cohort was stratified into 218 patients with RRMS and 81 patients with PPMS. All patients were unrelated and met the following inclusion criteria: diagnosed with MS according to the revised McDonald criteria (Thompson et al., 2018); clinical course of RR or PP MS (Lublin and Reingold, 1996); older than 18 years of age. Patients presenting clinically isolated syndrome or any other CNS diseases were not considered eligible for enrollment. Random recruitment was restricted to patients who gave written informed consent for their participation in the study as well as for DNA analysis.

The following data were collected for each patient: year of onset of MS; disease duration at the last follow-up; level of disability at the last follow-up according to the expanded disability status scale (EDSS) (Kurtzke, 1983); progression index (PI), which was calculated as the ratio EDSS/disease duration (Cendrowski, 1986). Moreover, a group of 619 unrelated healthy controls from the Sardinian Voluntary Bone Marrow Donor Registry, all aged at least 50 years at the time of enrollment, was recruited. This was done to minimize the probability of including subjects at risk of developing MS in the control group, given that most individuals are diagnosed with MS between the ages of 20 and 50 years (Vaughn et al., 2019).

All the 619 healthy controls and the 299 patients affected by MS originated from Sardinia at least two generations. This is to avoid bias due to the peculiar genetic background of the Sardinian population (Contu et al., 1992).

### 2.2. HLA alleles and KIR genotyping

Genomic DNA was extracted from peripheral blood mononuclear cells according to manufacturer’s instructions (QIAGEN, 2023).

All 918 samples from patients and controls were genotyped at high resolution for the alleles at *HLA-A*, *-B*, *-C*, and *-DRB1* loci using next-generation sequencing (NGS) AlloSeq Tx17 (CareDx) method based on Hybrid Capture Technology and performed on the Illumina platform. The data was analyzed using the AlloSeq Assign^®^ software (v.1.0.2).

The presence of 11 KIR genes (*KIR2DL1*, *KIR2DL2*, *KIR2DL3*, *KIR2DL5*, *KIR3DL1*, *KIR2DS2*, *KIR2DS3*, *KIR2DS4*, *KIR2DS5*, and *KIR3DS1*) in patients and controls was determined by typing genomic DNA through PCR with primers specific to each locus, following a previously reported method (Uhrberg et al., 1997; Gagne et al., 2002).

The HLA alleles and KIR of controls and patients are listed in Table 1.

**TABLE 1:** Immunogenetic and genetic parameters of the cohort used to build the models and number of values.

	Parameters	Number of values
Gender	Female, male	2
HLA-A*	01, 02, 03, 11, 23, 24, 25, 26, 29, 30, 31, 32, 33, 36, 66, 68, 69, 74	18
HLA-B*	03, 07, 08, 13, 14, 15, 18, 27, 35, 37, 38, 39, 40, 41, 44, 45, 47, 49, 50, 51, 52, 53, 55, 56, 57, 58, 73, 78	28
HLA-C*	01, 02, 03, 04, 05, 06, 07, 08, 12, 14, 15, 16, 17	13
HLA-DRB1*	01, 03, 04, 07, 08, 09, 10, 11, 12, 13, 14, 15, 16	13
KIR	2DL1, 2DL2, 2DL3, 2DL5, 3DL1, 2DS1, 2DS2, 2DS3, 2DS4, 2DS5, 3DS1	11

Note that some controls and patients show the same HLA parameters and the same KIR genes. In particular, this happens for:

• three couples patient/control
• one subset of three patients and one control
• one subset of two patients and four controls,

all with HLA-A*30, HLA-B*18, HLA-C*05, HLA-DRB1*03 in common, associated with MS susceptibility.

## 3. Classification models

### 3.1. Naïve Bayes and decision trees

In this work, a binary classification has been performed between healthy people (class controls = 0) and patients (class patients = 1) in order to evaluate the risk of developing MS. In this context, DT models, which belong to the supervised ML classification methods, have been applied.

Decision trees (DTs) are one of the oldest ML techniques referring to a hierarchical model of decisions and their consequences. The tree partitions a data set that contains examples belonging to different classes, two or more, associated to a label or a target value, which specifies the exact class. Each example is composed of a number of different and independent attributes, also called features. The rationale of DTs classifier is expressed by recursively partitioning of the feature space. Among all of ML techniques available to address the classification problems, such as for example neural networks (NNs) and support vector machines (SVMs), the main reasons that led us to the choice of DTs for this specific application are:

(1) DTs can handle both categorical and numerical input data;
(2) DTs are self-explanatory and, when compacted, they are also easy to interpret (Rokach and Maimon, 2015). This property is fundamental for the purpose of the work because it can be possible for medical professionals to comprehend the set of rules behind each decision;
(3) DTs can deal with datasets that may contain errors and missing values;
(4) DTs allow us to directly evaluate the contribution of each feature, which is essential to interpret the decision made.

In particular, the proposed prediction models follow the implementation adopted in the Matlab toolbox for statistics and machine learning (The MathWorks Inc, 2019).

A Naïve Bayesian (NB) classifier can be implemented as a benchmark for comparison to DTs model. Despite its simple structure, the NB algorithm generally provides very competitive results. Based on Bayes’ theorem (Mitchell, 1997), it assumes that each feature is independent and does not interact with each other, such that each feature independently and equally contributes to the probability of a sample to belong to a specific class.

More details on DT and NB models are reported in a Supplementary File.

Occasionally, to obtain a model with more accurate and reliable decisions than those of single models, ensemble learning can be adopted. Ensemble learning is a method that combines multiple models to produce a single model with improved results (Rokach and Maimon, 2015). A majority voting ensemble (MVE) combines the predictions from multiple models. In the case of classification, the MVE label is the label with the majority of votes. There are two approaches: hard voting and soft voting. Hard voting involves predicting the class label with the most votes. Soft voting sums the predicted scores for each class label and predicts the class label with the largest score.

### 3.2. Cross validation

Generally speaking, test performance gives an idea of how well a model will perform on unseen data. However, in case of limited data set, they can greatly vary depending on which observations were used in the training and testing sets. One way to avoid this problem is to create several models using a different training and testing set each time, then evaluating the performance as the average of all of the test performance. This general method is known as cross-validation and a specific form of it is known as leave-one-out (LOO) cross-validation. LOO adopts the following approach to evaluate a model: the dataset is split into a training set and a test set, using all but one observation as part of the training set. The model, built using only data from the training set, is used to predict the response value of the one observation left out and to calculate the performance. The process is repeated *k* times, where *k* is the total number of observations in the dataset. Then, the test performance is calculated as the average of all the *k* results.

Both in decision tree and Naïve Bayes classifiers, the optimal threshold for the posterior probability is normally obtained by the cross-validation procedure in correspondence to the optimal operating point of the receiving operating characteristic (ROC) curve (Fawcett, 2006).

## 4. Encoding of HLA

Data pre-processing and encoding are mandatory in order to improve efficiency. As reported in Table 1, some considered attributes are qualitative, i.e., gender (male, female) and genotype data like human leukocyte antigens–HLA (HLA-A*01, HLA-A*02 alleles, etc.). As the DT model can handle categorical variables, there is no need to use any encoding for gender, while for the genotype data, several encodings have been tested to feed the prediction model. Conversely, the 11 KIRs (2DL1, 2DL2, etc.) are encoded in binary form with *1* and *0* indicating the presence or absence of each KIR, respectively.

In Table 1, the HLAs are arranged in ascending order and their variants are only those present in the dataset. In humans, the HLA is a complex of genes residing on chromosome 6; considering that a human genome contains pairs of chromosomes, there are 2 copies of each HLA.

In this work, five different types of HLA data encoding, explained in the following, have been tested. For the sake of clarity, the different data encodings are reported (see Tables 2–6) referring to the haplotype example: HLA-A*01, HLA-A*68, HLA-B*08, HLA-B*35, HLA-C*04, HLA-C*07, HLA-DRB1*03, HLA-DRB1*04.

**TABLE 2:** Representation of the haplotype example without encoding (encoding 1).

Feature index	1	2	3	4	5	6	7	8
Feature name	HLA-A^1^*	HLA-A^2^*	HLA-B^1^*	HLA-B^2^*	HLA-C^1^*	HLA-C^2^*	HLA-DRB1^1^*	HLA-DRB1^2^*
Value	01	68	08	35	04	07	03	04

**TABLE 3:** Representation of the haplotype example for one-hot encoding 1 (encoding 2).

Feature index	1	…	30	…	56	…	62	…	89	…	104	…	114	…	128	…	131
Feature name	HLA- A^1^*01	…	HLA- A^2^*68	…	HLA- B^1^*08	…	HLA- B^2^*35	…	HLA- C^1^*04	…	HLA- C^2^*07	…	HLA-DRB1^1^*03	…	HLA-DRB1^2^*04	…	HLA-DRB1^2^*09
Value	1	0	1	0	1	0	1	0	1	0	1	0	1	0	1	0	0

**TABLE 4:** Representation of the haplotype example for one-hot encoding 2 (encoding 3).

Feature index	1	…	17…	25…	42…	47…	54…	68…	69…	72
Feature name	HLA- A*01	…	HLA- A*68…	HLA- B*08…	HLA- B*35…	HLA- C*04…	HLA- C*07…	HLA-DRB1*03…	HLA-DRB1*04…	HLA-DRB1*09
Value	1	0	10	10	10	10	10	10	10	0

**TABLE 5:** Representation of the haplotype example for the combination without encoding (encoding 4).

Feature index	1	2	3	4
Feature name	HLA-A*	HLA-B*	HLA-C*	HLA-DRB1*
Value	(01, 68)	(08, 35)	(04, 07)	(03, 04)

**TABLE 6:** Representation of the haplotype example for the combination 1H encoding (encoding 5).

Feature index	1	…	16	…	261	…	318	…	391	…	419
Feature name	HLA-A*(01, 01)	…	HLA-A*(01, 68)	…	HLA-B*(08, 35)	…	HLA-C*(04, 07)	…	HLA-DRB1*(03, 04)	…	HLA-DRB1*(13, 15)
Value	0	0	1	0	1	0	1	0	1	0	0

In the following, the five data coding are reported. Note that, the superscripts 1 and 2 refer to the HLA allele variants in the two chromosomes.

(1) Without encoding: the information about the HLAs is maintained as it is. Thus, the dataset contains 8 categorical features (see Table 2). The value of the attribute is represented by a letter and a number, e.g., HLA-A^1*^01, or HLA-A^2*^68, which means that the class of the HLA is A, and the alleles are, respectively, 01 and 68. In this way, the single HLA feature can take several values; thus, HLAs assume higher importance with respect to KIRs that are binary variables. Since the HLA alleles in the chromosomes are arranged in ascending order, the information on the first one contains some information about the second one. For example, if the feature value HLA-B^1*^08 is present, the values of the feature HLA-B^2*^ cannot be lower than 08, i.e., features HLA-B^2*^03 or HLA-B^2*^07 cannot appear. This consideration is valid also for the following encoding 2.
(2) One-hot (1H) encoding 1: in one-hot encoding, a feature with *d* possible values is transformed into *d* binary features. In this case, there is one feature for each allele variant (equal to 1 or 0 for presence or absence) and the HLA allele variants in the two chromosomes supply two different features. The names of the features are represented by a letter and a number. Referring to the proposed haplotype example, HLA-A^1*^01 assumes the value 1, as well as HLA-A^2*^68, whereas the other features HLA-A^1*^ and HLA-A^2*^ have the value 0. And so also for the other HLAs. This leads to a total number of features equal to 131 in the available dataset and, for each instance, only 8 features assume a value equal to 1, whereas the other 123 are zeros.
(3) One-hot encoding 2: every attribute value related to each one of the 4 HLAs (A, B, C, DRB1) is encoded with one-hot encoding, thus each HLA allele supplies one feature. This leads to a number of features equal to 72 in the available dataset (see Table 1). With this encoding, for each instance, at most 8 features assume a value equal to 1.
(4) Combination without encoding: in this encoding, the HLA alleles related to the same gene are combined to form one single feature; the attribute value is represented by a letter and two numbers, i.e., HLA-C*(04, 07), which means that the HLA belongs to the type C and the numbers are related to the allele variants in the two chromosomes. This leads to 4 features, one for each HLA type, i.e., *HLA-A*, *HLA-B*, *HLA-C*, and *HLA-DRB1* and a number of possible values equal to 419. In this way, the single HLA feature can take several values; thus, like in the first encoding, HLAs assume higher importance with respect to KIRs that are binary variables.
(5) Combination 1H encoding: the previous combination of HLAs is encoded with one-hot encoding, which means that the 419 values are transformed into 419 features that can only have two possible attribute values, 0 or 1.

Tables 7, 8 show the number of features and some examples of possible values for data representations without and with encoding, respectively. For more details about attribute values refer to Table 1.

**TABLE 7:** Number of features, possible values and examples for data representations without encoding.

Data represen-tation	Number of HLA features	Number of values									Attribute value
		HLA-A*		HLA-B*		HLA-C*		HLA-DRB1*		**Total**	
1−without encoding	8	14	18	24	25	12	12	13	13	131	HLA-A*01, HLA-A*11, HLA-A*02, etc.
4−combination w/o encoding	4	95		174		79		71		419	HLA-A*(01, 01) HLA-A*(01, 11), HLA-A*(02, 32), etc.

**TABLE 8:** Number of features, possible values and examples for data representations with one-hot encoding.

Data represen-tation	Number of HLA features	Total number of values per feature	Attribute value
2−1H encoding 1	131	2	0, 1
3−1H encoding 2	72	2	0, 1
5−combination 1H encoding	419	2	0, 1

Generally speaking, different encodings produce different trees and assign different weights to their features. In particular, when there are features with a large number of values on which the tree can split, as for HLAs in encoding 1 and 4, the tree can grow in both directions. Moreover, these features will be given more importance than the binary features (KIR). Typically, these encodings produce the best results but are difficult to understand. On the contrary, when there are a few options for splitting, the decision trees result very sparse. The situation gets worse when features have a small number of values. One-hot encoding (encoding 2, 3, 5) falls in this category with just two values. In this case, the trees generally tend to grow in one direction, the one with zeroes in the variables. Moreover, using these last encodings, the reliability of the Gini’s index for the choice of the root node is very weak, since all the single conditions have low probability, and the impurity gain is very low.

## 5. Performance indexes

In the following section, performance of the proposed classifiers were tested on the database described in Section “2.1. Patients and control recruitment” by using the following performance indexes: TPR (true positive rate, also known as sensitivity or recall), which measures the ability of the model to correctly identify patients (people affected by MS); TNR (true negative rate, also known as specificity) which measures the ability of the model to correctly identify controls (healthy people); BA (balanced accuracy), suitable for imbalanced datasets since it is low if the model only predicts accurately for the majority class in the dataset, which is the arithmetic mean of sensitivity and specificity. Moreover, positive predictive value (PPV) and negative predictive value (NPV) allow to clinically say how likely a patient has or has not a specific disease. PPV and NPV have a strict dependence on prevalence (Prev), most commonly described as the percentage of people with the disease in a specified population. Generally speaking, as the prevalence increases, the PPV also increases but the NPV decreases. Similarly, as the prevalence decreases the PPV decreases while the NPV increases. More details on these performance indexes are reported in a Supplementary File.

Another performance index is the area (AUC) under the ROC curve (Fawcett, 2006).

Moreover, risk *R* of MS is defined as the difference between the percent of patients and the percentage of controls with a certain combination of HLAs.

## 6. Results

Given the limited size of the dataset (918 instances of patients and controls), splitting the data in training and test set has certain limitations. Indeed, when the dataset is small, the method is prone to high variance. To deal with this issue, 100 test sets of 60 instances have been created, each one composed by 30 instances of class “*patients*” and 30 instances of class “*controls.*” When creating the 100 test sets, each couple of patients and each couple of controls have been equally represented. For each test set, a model has been trained on the remaining 858 instances, 269 of class “*patients*” and 589 of class “*controls*.” Then, the prediction performance has been evaluated by averaging the performance of the 100 models.

In order to estimate the generalization performance, LOO cross-validation is applied. It supplies an estimate of the generalization performance of a model trained on *k-1* instances of data which is a slightly pessimistic estimate of the performance of a model trained on *k* instances.

Several tests have been performed to optimize the hyperparameters of the models, such as the number of splits, the minimum number of samples required to be a leaf node (minimum leaf size), the impurity index, the number of features to consider when looking for the best split and the prior probabilities associated with the classes. The models showing the best BA in LOO are selected.

Note that, after the first tests, the gender’ feature has been discarded since strongly biases the classifications (e.g., encoding 1 and 2 classified all the women as “patients”).

Table 9 shows the average performances of the 100 models, on training, LOO and test set.

**TABLE 9:** Average performance on the 100 tests on training, LOO and test set.

	1−without encoding	2−1H encoding 1	3−1H encoding 2	4−combination w/o encoding	5−combination 1H encoding
**BA (%)**					
Training	66.65	59.44	58.38	81.61	63.61
LOO	57.36	59.42	57.31	58.77	62.03
Test	56.55	59.43	57.02	58.45	62.32
**TPR (%)**					
Training	61.72	70.91	64.39	90.01	68.09
LOO	58.16	70.86	62.87	61.93	66.49
Test	57.93	70.87	62.37	61.60	66.77
**TNR (%)**					
Training	61.72	47.98	52.38	73.21	59.13
LOO	56.57	47.98	51.76	55.61	57.58
Test	55.17	48.00	51.67	55.30	57.87

As it can be noticed, performance on LOO and test sets are close, even if, as expected, worse than those obtained in the training set. This result suggests that, to obtain a final model, it is reasonable to train a DT on the whole dataset with LOO cross validation. Its LOO performance, although representing an optimistic estimate of performance on the test set, will not be too far from it.

### 6.1. Decision trees results

In order to obtain the final model, a DT is trained with the whole dataset and LOO cross-validation.

Table 10 shows the performance of the final five trained models with LOO procedure. Results are reported for the whole data set, and for men/women subsets. It can be seen that LOO and training performances are close, confirming the generalization capabilities of the DT. Moreover, performances for women are worst for all but encoding 2.

**TABLE 10:** Performance for the training and leave-one-out procedure.

		Encoding														
		**1−without encoding**			**2−1H encoding 1**			**3−1H encoding 2**			**4−combination w/o encoding**			**5−combination 1H encoding**		
	**Gender**	**M**	**W**	**All**	**M**	**W**	**All**	**M**	**W**	**All**	**M**	**W**	**All**	**M**	**W**	**All**
BA %	Training	68.68	64.59	66.64	69.29	68.27	68.70	65.32	61.47	63.26	80.52	78.26	79.56	66.24	64.40	65.62
	LOO	66.42	61.20	63.64	65.77	66.18	66.00	65.32	61.47	63.26	66.74	62.61	64.46	64.32	63.14	64.05
TPR %	Training	71.84	71.43	71.57	69.90	68.88	69.23	68.93	66.84	67.56	84.47	86.22	85.62	66.02	69.39	68.23
	LOO	67.96	65.31	66.22	66.99	67.35	67.22	68.93	66.84	67.56	68.93	65.82	66.89	64.08	67.86	66.56
TNR %	Training	65.51	57.76	61.71	68.67	67.66	68.17	61.71	56.11	58.97	76.58	70.30	73.51	66.46	59.41	63.00
	LOO	64.87	57.10	61.07	64.56	65	64.78	61.71	56.11	58.97	64.56	59.41	62.04	64.46	58.42	61.55
Min leaf size		50			11			20			20			11		

Table 11 reports the results separately for RRMS and PPMS cases. For encoding 1 and 4, results for PPMS cases are slightly better. Note that most patients present the RRMS variant.

**TABLE 11:** True positives (TP), false negatives (FN) and true positive rate (TPR) for RRMS and PPMS patients.

		Encoding				
	**Indexes**	**1−without encoding**	**2−1H encoding 1**	**3−1H encoding 2**	**4−combination w/o encoding**	**5−combination 1H encoding**
All (299)	TP	198	201	202	200	199
	FN	101	98	97	99	100
	TPR %	66.22	67.22	67.56	66.89	66.56
RRMS (218)	TP	145	146	149	144	143
	FN	73	72	69	74	75
	TPR %	66.51	66.97	68.35	66.06	65.60
PPMS (81)	TP	53	55	53	56	56
	FN	28	26	28	25	25
	TPR %	65.43	67.90	65.43	69.14	69.14

The five encodings have very close performance, with encodings 1 and 4 showing best values of BA on LOO. As expected, despite similar performance, they produce different trees and assign different weights to their features. In encoding 1 and 4 the tree grows in both directions favoring HLA features which present several values and discarding the binary KIR features. The decision trees’ rules generated from these encodings involve multiple alternative conditions, making their interpretation challenging despite yielding optimal results.

In one-hot encoding (encodings 2, 3, 5), when there are a few options for splitting, the decision trees result very sparse. The trees grow in the direction of the zero variables.

**FIGURE 1:**
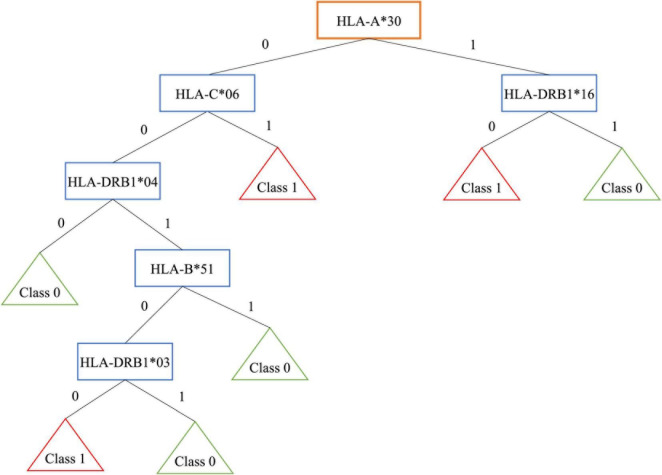
Decision tree for encoding 3.

As an example, Figure 1 shows the obtained tree for encoding 3 that is the most interpretable even if it is not the best one. Patients are grouped in 3 leaves, e.g., one of them is associated to people with HLA-A*30 without HLA-DRB1*16 (*R* = 16.07%, 121/299 patients and 151/619 controls).

On the contrary, controls are grouped in 4 leaves. One of them is associated to people without HLA-A*30, HLA-C*06, HLA-DRB1*04 (*R* = −21.35%, 69/299 patients and 275/619 controls).

Note that, the well-known extended haplotype HLA-A*30, HLA-B*18, HLA-C*05, HLA-DRB1*03, shows a risk *R* = 11% with 104/299 patients and 146/619 controls, while the extended haplotype HLA-A*02, HLA-B*58, HLA-C*07, HLA-DRB1*16 shows a negative risk *R* = −6.73%, with 19/299 patients and 81/619 controls.

In the first haplotype, the presence of HLA-A*30 belonging to the risky haplotype is associated with the absence of HLA-DRB1*16 included in the protective haplotype. This results in a higher risk. The encoding 5, where the HLA couple is supplied as input, identifies several classes of patients, for example some associated to the well-known HLA susceptibility allele HLA-DR*03:

• HLA-DRB1*{03, 03}; *R* = 9%, with 37/299 patients and 22/619 controls.
• HLA-DRB1*{03, 15}; *R* = 3%, with 12/299 patients and 8/619 controls.

### 6.2. Multiple classifier system

Individual decision trees suffer from variances, i.e., small variations of training data set may lead to a disproportional modification of the model. Moreover, greedy algorithms cannot guarantee to return the globally optimal decision tree.

These disadvantages can be mitigated by training multiple trees. Thus, multiple classifiers or random forests (RFs) could address these issues (Rokach and Maimon, 2015). Since the limited dataset is a problem for bootstrap resampling performed by RFs, this technique has been discarded and only the simplest multiple classifier system (MCS) has been considered. Combining the prediction of several classifiers reduces variance and enhances generalization. Hard classification voting has been adopted as decision rule. In this case, predictions are the majority vote of contributing models. The MCS improves classification accuracy with respect to the single trees.

**FIGURE 2:**
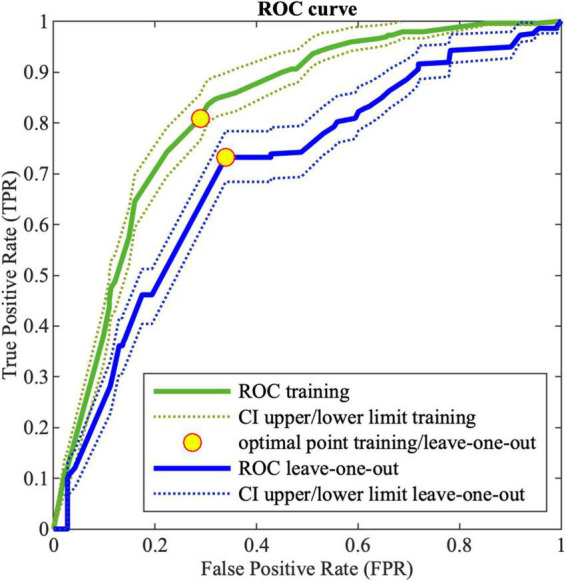
Receiving operating characteristic (ROC) curve for training (green) and LOO (blue) for MCS; the dotted lines (green and blue for training and leave-one-out, respectively) corresponds to the 95% confidence interval; the yellow points correspond to the optimal threshold.

Figure 2 shows the ROC curve obtained by the MCS on LOO. The best obtained performances on training data and LOO are shown in Table 12, where also the *AUC* is reported. As it can be noticed the ensemble of trees shows enhanced results than single models for all the performance indices. For the sake of comparison, Table 12 shows also the results obtained by implementing a MCS from NB models, trained with the same database and coding used for DTs.

**TABLE 12:** MCS performance on DTs and NB classifiers for the training data.

	Training		LOO	
	**DTs**	**NB**	**DTs**	**NB**
TP	242	208	219	176
FN	57	91	81	123
TPR %	80.94	69.57	73.24	58.86
TN	440	409	409	368
FP	179	210	210	251
TNR %	71.08	66.07	66.07	59.45
BA %	76.01	67.82	69.66	59.16
AUC %	82.02	75.20	69.53	62.13

The MCS constructed by means of a DT ensemble presents better performances with respect to all the indexes.

It is important to notice that, due to the fact that there are some controls and patients that show the exact same genotype, as mentioned in 2.3, the algorithm makes inevitable errors in the classification. The error resulting from such ambiguity in the input data consists of 1 *FN* and 7 *FPs*.

The performances achieved with the MCS system appear to be very promising with a TPR greater than 73%. This tool would enable clinicians to identify individuals at higher risk among the relatives of MS patients, who require more frequent and careful follow-up.

Multiple sclerosis (MS) is a rare disease, by definition its prevalence is very low. So, when a diagnostic test will be used in the low-prevalence population, it will have a poor PPV. In contrast, with a good screening test the NPV will be high. To increase the PPV of the test, it could be targeted to those at high risk of developing the disease, based on considerations such as demographic factors, medical history, etc. In this case, the selected population would be characterized by higher prevalence with respect to the whole population.

**FIGURE 3:**
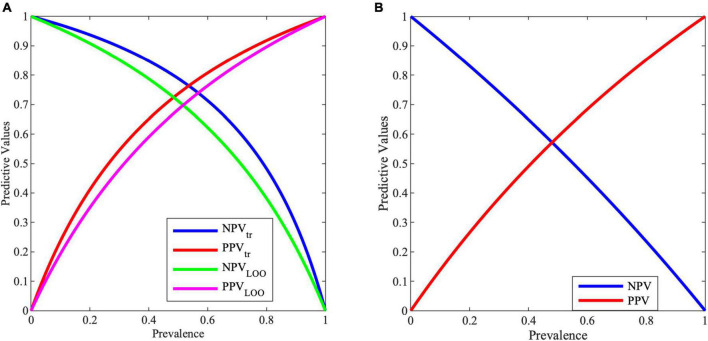
Negative predictive value (NPV) and positive predictive value (PPV) for varying prevalence values from **(A)** MCS model, **(B)** entropy work.

Figure 3A shows the variation of PPV and NPV values with the prevalence, i.e., with the selected screened population. As it can be noticed, with a prevalence of 0.5, PPV and NPV are for training (tr) 74 and 79%, while for validation (LOO) 68 and 71%, respectively.

Unfortunately, contrarily to individual DTs, the reasons behind the MCS decision cannot be described as a rule. As an example, let us consider the answer of the MCS for instances (104 patients and 146 controls) with the extended haplotype HLA-A*30, HLA-B*18, HLA-C*05, HLA-DRB1*03, associated to MS. In this case, the system shows a TPR of 89%, recognizing 93 patients over 104, and a TNR of 40%, correctly identifying 59 controls over 146.

Thus, in the face of 11% of misclassified patients, the MCS is able to correctly identify 40% among the 146 controls with the extended haplotype, which would be all considered at high risk of developing MS when considering only the information coming from the presence of the extended haplotype. It is likely that the performance of the MCS decision system could be further improved by extending the HLA analysis to other loci in addition to the 4 currently considered (HLA-A, HLA-B, HLA-C, and HLA-DRB1).

### 6.3. Comparison between entropy-based risk test and decision trees model

In order to compare the performances of the methods, the same indexes used for decision trees were calculated for the entropy-based model. Since the well-known indexes, as TPR, TNR, etc., are designed for binary problems, then the three degrees risk entropy model was reduced to a two-classes merging high and medium risk into one single class. Thus, the positive class is made by high and medium risk and the negative class by low risk. This ensures better performances with respect to merging low and medium risk classes into one class.

It can be noticed that the entropy-based model results are calculated on the same dataset of controls and patients used to develop the risk model (the so-called training set). Instead, for the DTs model, to avoid the risk of overfitting, performances are computed both for training set and LOO validation set. Performances on validation set are a better estimate of the model behavior in case of unseen data.

Considering the performances related to the studies, it is possible to notice that the performances for controls are similar, with a TNR (ability to correctly detect healthy controls) for the entropy study of 70.27% and for the MCS of 71.08 and 66.07% on training and validation data, respectively. Instead, for MS patients, the performances are vastly different with TPR (ability to correctly detect people affected by MS) for the entropy study of 42.96% and for the MCS of 80.94 and 73.24% on training and validation data, respectively.

Predictive values are important information for clinicians. In the proposed case, with a prevalence of 0.5, PPV and NPV are for training 73.68 and 78.86%, while for validation 68.34 and 71.17%, respectively. These results in an improvement with respect to the entropy work, where for a prevalence of 0.5, PPV and NPV reach a value of 59.10 and 55.20%, respectively, as shown in Figure 3B.

## 7. Discussion and future work

Multiple sclerosis (MS) is a complex autoimmune disease affecting the CNS. While genetics plays a role in the development of MS (Goodin et al., 2021), environmental factors such as Epstein-Barr virus (EBV), vitamin D deficiency, cigarette smoking, age, and gender are also identified as important risk factors (Handel et al., 2010; Miljković and Spasojević, 2013; Taylor et al., 2015; Thorley-Lawson, 2015; Olsson et al., 2017; Parnell and Booth, 2017).

Early diagnosis and treatment are crucial, and immunotherapy using immunomodulatory drugs (IMDs) is currently the primary approach to slow its progression (Frau et al., 2018; Pachner, 2022). In recent years, the application of artificial intelligence (AI) in healthcare (Davenport and Kalakota, 2019) has shown significant potential, particularly in the analysis of medical images created by MRI. However, the application of AI methods to the analysis of MS images still faces several challenges (Hosny et al., 2018; Prevedello et al., 2019; Bonacchi et al., 2022).

The new era of genomic medicine may open new avenues for developing accurate and reliable diagnostic tools based on genetic sequencing analysis and ML approaches (Brittain et al., 2017). Such tools may help to identify new biomarkers for early detection of disease onset and progression as well as the development of novel drugs to cure different neurological disorders (Chang et al., 2021).

To date, there are more than a hundred relevant published papers, with more than half of these involving the detection and segmentation of MS lesions for quantitative analysis. However, only a few studies have applied AI/ML tools to clinical genetic and environmental risk factors to predict the development and progression of MS (Hartmann et al., 2021). This is partly due to the fact that the majority of MS’s heritable component has yet to be discovered. While large-scale and collaborative genome-wide association studies (GWAS) and targeted functional studies continue to uncover new risk loci (Li et al., 2021), the risk linked to *HLA* alleles remains the highest among other susceptibility genetic variants (De Silvestri et al., 2019; Lorefice et al., 2019). In this context, it is worth noting that the proposed method shares similarities with studies employing the Polygenic Risk Score (PRS) approach, given its potential to consider and incorporate the information from *HLA* alleles, which have been consistently shown to possess the highest risk among other genetic variants associated with susceptibility to MS (Hone et al., 2021; Breedon et al., 2023).

The current study is the first attempt to use AI/ML to discriminate subjects at higher risk of susceptibility to MS based on their immunogenetic characteristics HLA and KIR.

This study used an ensemble learning of DTs, a supervised ML algorithm, applied to HLA and KIR. The most interesting aspect of the results, obtained through leave-one-out cross-validation of the MCS (as shown in Table 12), is the system’s ability to correctly identify 73.24% of MS patients and 66.07% of healthy subjects. This performance makes the system a potential tool to help clinicians to monitor the relatives of MS patients to discriminate subjects with a higher risk of predisposition to the disease. The main limitation is the percentage of MS patients, 26.76%, that the algorithm cannot identify.

Furthermore, it was found that the MCS of DTs produces better results, when compared with the entropy model (Melis et al., 2019) and with the MCS of NB models, in the analysis of HLA/KIR genetic combinations.

While the TNR (ability to correctly detect healthy controls) of the two analysis methods is comparable (66.07 and 70.27%, for MCS and entropy model, respectively), the TPR of the MCS (73.24%) is significantly higher than that of the entropy model (42.96%).

The complex pathogenetic mechanisms underlying MS, as with all multifactorial diseases, derive from the interplay between genetic predisposition and environmental factors. Each individual has a well-defined epigenetic profile, given by the combination of all these factors. These profiles, in combination with HLA and KIR, could potentially improve the predictive performance of the MCS.

The major flaw of applying a ML technique to this particular dataset is the size of the dataset itself; considering the number of features of the problem, i.e., the immunogenetic parameters, it is essential that the size of the dataset is adequate. In fact, to avoid the curse of dimensionality problem, it is necessary to have an amount of well-representative training data. In general, as the number of features grows, the amount of data needed to generalize accurately grows exponentially. In this paper some strategies have been adopted to take into account the small sample size.

While the preliminary results appear promising, further investigation is warranted using larger sample sizes especially in different ethnic groups that are less homogeneous than the population of Sardinia. Such research would aid in a more comprehensive understanding of the potential clinical applications of the MCS.

Although MS can manifest with various clinical characteristics, disease courses, and etiologies, its pathogenic mechanism is substantially immunological. Therefore, the integration of a larger set of immunogenetic markers, obtained by typing the non-classical MHC alleles (*HLA-G*, *HLA-E*, *HLA-F*, *MIC-A*, *MIC-B*) along with alleles of their specific lymphocyte receptors (*ILT2*, *ILT4*, *NKG2A*, etc.), is likely to enhance the results obtained in this pilot study in terms of predictive capacity and accuracy.

This approach may provide valuable insights into the disease’s pathogenesis and help to refine the predictive model, ultimately contributing to improved personalized risk assessments and treatment strategies for individuals affected by MS. As this study serves as a pilot investigation, the inclusion of further immunogenetic markers in future research could significantly enhance the predictive capabilities and shed light on the intricate immunological aspects underlying MS.

In conclusion, the use of the MCS has demonstrated to be an accurate and reliable diagnostic tool capable of supplying promising results in discriminating subjects at a higher risk of developing the disease based on their immunogenetic characteristics.

## Data availability statement

The datasets presented in this study can be found in online repositories. The names of the repository/repositories and accession number(s) can be found below: https://www.ncbi.nlm.nih.gov/, PRJNA987444.

## Ethics statement

The studies involving humans were approved by the Ethics Committee of the Cagliari University Hospital. The studies were conducted in accordance with the local legislation and institutional requirements. The participants provided their written informed consent to participate in this study.

## Author contributions

MP, FP, BC, and AF contributed to conception and design of the study. EC and JF enrolled the patients. FL, SM, RL, and SG organized the database with immunogenetic parameters. MP trained the classification models. MP, FP, BC, AF, SM, RL, and SG wrote the first draft of the manuscript. All authors contributed to manuscript revision, read, and approved the submitted version.

## Supplementary material

Supplementary Data Sheet 1 Details on classification models.

Supplementary Data Sheet 2 Details on performance indexes.
