# Supplementary material for: Decision trees to evaluate the risk of developing multiple sclerosis
Source: Front Neuroinform. 2023 Aug 15;17:1248632. doi: 10.3389/fninf.2023.1248632 (PMC10465164; doi:10.3389/fninf.2023.1248632)
Supplement: Supplementary Data Sheet 1 — Details on classification models. [file Data_Sheet_1.docx]

**Decision Trees**

The DTs consist of nodes with incoming and outcoming edges. The first and only node that has no incoming edges is known as “root”; all other nodes have one incoming edge. Nodes with outcoming edges are called “branch” nodes, otherwise they are “leaves” or “terminal” nodes. Once the DT is trained, new instances are classified by navigating them from the root of the tree down to a leaf^[[1]](#footnote-1)^.

The decision-making process of a DT is similar to rule induction. In fact, driving the edges from the root node, through internal nodes, up to the leaves, it is possible to transform the path into a rule. The goal of recursive partitioning is to divide the feature space in a way that the observations within a terminal node are as similar as possible. In order to perform that, a splitting criterion is needed; in this work an internal node is split according to the value of a single attribute, i.e., a univariate splitting criterion^1^. The rationale is to select the attribute that is most useful for the particular classification problem; in general, the best selected split is the one that determines the most homogeneous sub-nodes. One node is “entirely pure” if it contains only examples from a single class. Intuitively, the more the purity of the nodes, the more the homogeneity. There are several possible indexes that aim to calculate the impurity at the nodes, like Entropy Index, Gini’s Diversity Index, Twoing Rule, etc.,^1^. Among these, the Gini’s Diversity Index (gdi) is one of the most common splitting criteria; given a node *n* and estimated the class *q_i_* posterior probability Pr(*i*),  i = 1, …, Q, where Q is the number of classes, gdi is defined as^[[2]](#footnote-2)^:

$gdi=1-\sum_{i=1}^{Q} \Pr\left(i\right)^{2}=\sum_{i=1}^{Q} \Pr\left(i\right)\left(1-\Pr\left(i\right)\right)$ (1)

The posterior probabilities can be also weighted, considering the importance of each class, by setting suitable prior probabilities.

Gini’s Diversity Index varies between values 0 and 1 – 1/Q, where 0 expresses the purity of classification and 1 – 1/Q corresponds to a random distribution of samples across the classes.

Restricting to a binary classification problem, if S is a subset of the training set reaching a particular node *n* of the tree, Pr(1|n) and Pr(0|n) the posterior probabilities of the classes 1 and 0 at the node *n*, respectively, the Gini’s Diversity Index is:

$gdi=1-\Pr\left(1|n\right)^{2}-\Pr\left(0|n\right)^{2}=2\Pr\left(1|n\right)\Pr\left(0|n\right)$ (2)

In general, there are two types of algorithms to select the best predictor for each node. In the first type, the selection implies the choice of a splitting criterion which takes into account the purity of the split. The best predictor for each branch node is then selected, from all possible predictors, as the one that maximizes the difference between the impurity of the node and the average impurity of its children nodes^2^. Instead, in the second type, a statistical test, that assesses that there is no association between variables, is performed. In this case, the best split predictor is the one that minimizes the significant *p*-value (less than 0.5) between each predictor and the response variable (curvature test^[[3]](#footnote-3)^), or the one that minimizes both the *p*-value between each predictor and the response and between each pair of predictor and the response (interaction-curvature test^[[4]](#footnote-4)^).

The impurity measure of branch nodes related to a given attribute can also be used for the estimation of the attribute importance as a predictor; the importance is estimated by averaging, among branch nodes making use of that feature, the differences between the impurity of each branch node and the average impurity of its children nodes.

DTs are considered greedy algorithms, which means that they will continue to split until they have a node that is as pure as possible; this can lead to very deep classification trees that can often result in an overfitting on the training data. In order to control the tree depth, a maximum value of the number of splits or a minimum number of leaf node observations should be imposed.

Once the DT is constructed, in order to reach a decision on a test instance, it is sufficient to follow the conditions in the tree until a leaf is reached. Based on the training set distribution, a posterior probability is assigned to the leaf, and a score is assigned to the test sample. If the score is above (or below) a certain threshold, the test instance is assigned to the positive (or negative) class.

**Naïve Bayes**

From Bayes’ theorem follows:

$\Pr\left(q_{i}|x\right)=\frac{\Pr\left(x|q_{i}\right)\Pr\left(q_{i}\right)}{\Pr\left(x\right)}=\frac{\Pr\left(x|q_{i}\right)\Pr\left(q_{i}\right)}{\sum_{k} \Pr\left(x|q_{k}\right)\Pr\left(q_{k}\right)}$ (3)

where: Pr(*q_i_*|*x*) is the posterior conditional probability of a class label *q_i_* given a set *x* of predictors; Pr(*x*|*q_i_*) is the conditional probability of *x* given *q_i_*, calculated as the product of the conditional probabilities of each predictor given the class label; Pr(*q_i_*) is the prior probability of each class label, calculated on the basis of the frequency of that label in the training set. The NB algorithm consists normally in calculating the conditional probability Pr(*q_i_*|*x*) for each class by using Bayes’ theorem and then choosing the class label with the highest probability.

1. Rokach, L. & Maimon, O. Data mining with decision trees: theory and applications. (World Scientific, 2015). [↑](#footnote-ref-1)
2. Classification and regression trees. (Chapman & Hall/CRC, 1998). [↑](#footnote-ref-2)
3. Loh, W.-Y. & Shih, Y.-S. Split selection methods for classification trees. Stat. Sin. 7, 815–840 (1997). [↑](#footnote-ref-3)
4. Loh, W.-Y. Regression trees with unbiased variable selection and interaction detection. Stat. Sin. 12, 361–386 (2002). [↑](#footnote-ref-4)
