# Supplementary material for: Decision trees to evaluate the risk of developing multiple sclerosis
Source: Front Neuroinform. 2023 Aug 15;17:1248632. doi: 10.3389/fninf.2023.1248632 (PMC10465164; doi:10.3389/fninf.2023.1248632)
Supplement: Supplementary Data Sheet 2 — Details on performance indexes. [file Data_Sheet_2.docx]

# Performance indexes

Performance of the proposed classifiers has been tested by means of the following indexes:

$TPR=\frac{TP}{P}=\frac{TP}{TP+FN}$ (1)

$TNR=\frac{TN}{N}=\frac{TN}{TN+FP}$ (2)

$BA=\frac{TPR+TNR}{2}$ (3)

$PPV=\frac{TPR\cdot Prev}{TPR\cdot Prev+\left(1-TNR\right)\cdot\left(1-Prev\right)}$ (4)

$NPV=\frac{TNR\cdot\left(1-Prev\right)}{TNR\cdot\left(1-Prev\right)+\left(1-TPR\right)\cdot Prev}$ (5)

where $P$ is the actual number of Positives, i.e., number of *patients* in the set, $TP$ is the number of True Positives, i.e., the number of *patients* correctly classified, $N$ is the actual number of Negatives, i.e., the number of *controls* in the set, $FN$ is the number of False Negatives, i.e., the number of *patients* wrongly classified as *controls*; $TN$ is the number of True Negatives, i.e., the number of *controls* correctly classified, and $FP$ is the number of False Positives, i.e. the number of *controls* wrongly classified as *patients*; *Prev* is the Prevalence and is most commonly described as the percentage of people with the disease in a specified population.

In (1) $TPR$ is the True Positive Rate, also known as sensitivity or recall, and measures the ability of the model to correctly identify *patients* (people affected by MS).

In (2) $TNR$ is the True Negative Rate, also known as specificity, and measures the ability of the model to correctly identify *controls* (healthy people).

In (3) $BA$ is the Balanced Accuracy and represents the arithmetic mean of sensitivity and specificity.

In (4) PPV is the Predictive Positive Value and indicates the probability that following a positive test result, that individual will truly have that specific disease.

In (5) NPV is the Negative Predictive Value and indicates the probability that following a negative test result, that individual will truly not have that specific disease.
